# Supplementary material for: Inhibitory and synergistic effects of volatile organic compounds from bat caves against Pseudogymnoascus destructans in vitro
Source: mSystems. 2025 Dec 18;11(1):e00903-25. doi: 10.1128/msystems.00903-25 (PMC12817938; doi:10.1128/msystems.00903-25)
Supplement: Supplemental Material — Supplemental figures and tables. [file msystems.00903-25-s0001.docx]

**Inhibitory and synergistic effects of volatile organic compounds from bat caves against *Pseudogymnoascus destructans in vitro***

Zihao Huang ^1^, Shaopeng Sun ^1^, Yihang Li ^1^, Zizhen Wei ^1^, Mingqi Shen ^1^, Jiaqi Lu ^1^, Keping Sun ^2^, Zhongle Li ^1,3,*^, Jiang Feng ^1,3,*^

^1^ College of Life Science, Jilin Agricultural University, Changchun 130118, China.

^2^ Jilin Provincial Key Laboratory of Animal Resource Conservation and Utilization, Northeast Normal University, Changchun 130117, China.

^3^ Jilin Provincial International Cooperation Key Laboratory for Biological Control of Agricultural Pests, Changchun 130118, China.

*Corresponding authors, Email: Zhongle Li, lzy1514316@126.com; Jiang Feng, fengj@nenu.edu.cn

**
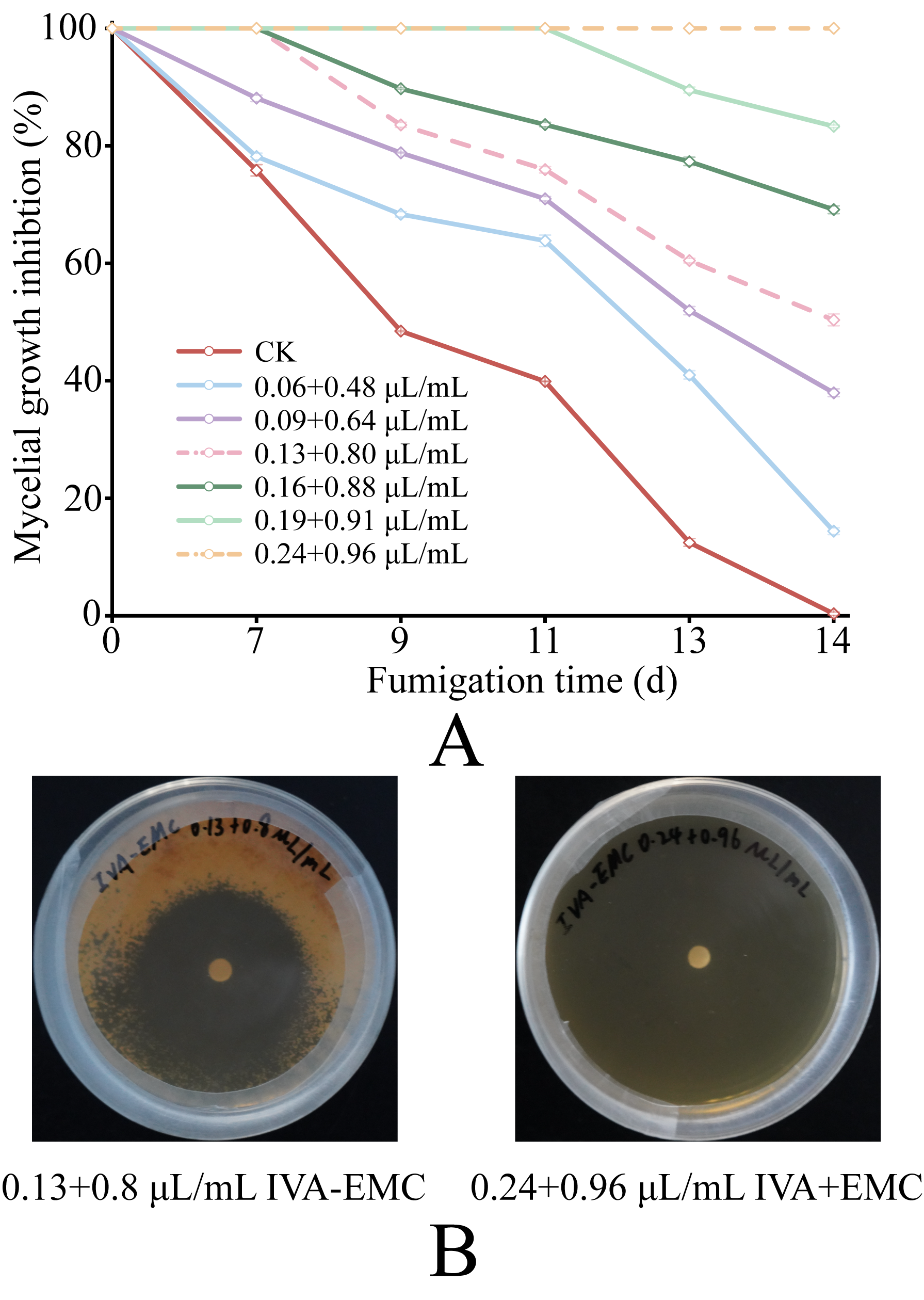
**

**Fig. S1.** Vapor-phase inhibition of *P. destructans* by IVA-EMC.(A) Gradient concentration assays of IVA-EMC after 14 d vapor-phase exposure. Representative plates showing mycelial growth at the IC_50_ and MIC levels.

**

**

**Fig. S2.** Comparison of RNA-seq results of representative DEGs with RT-qPCR results.

**
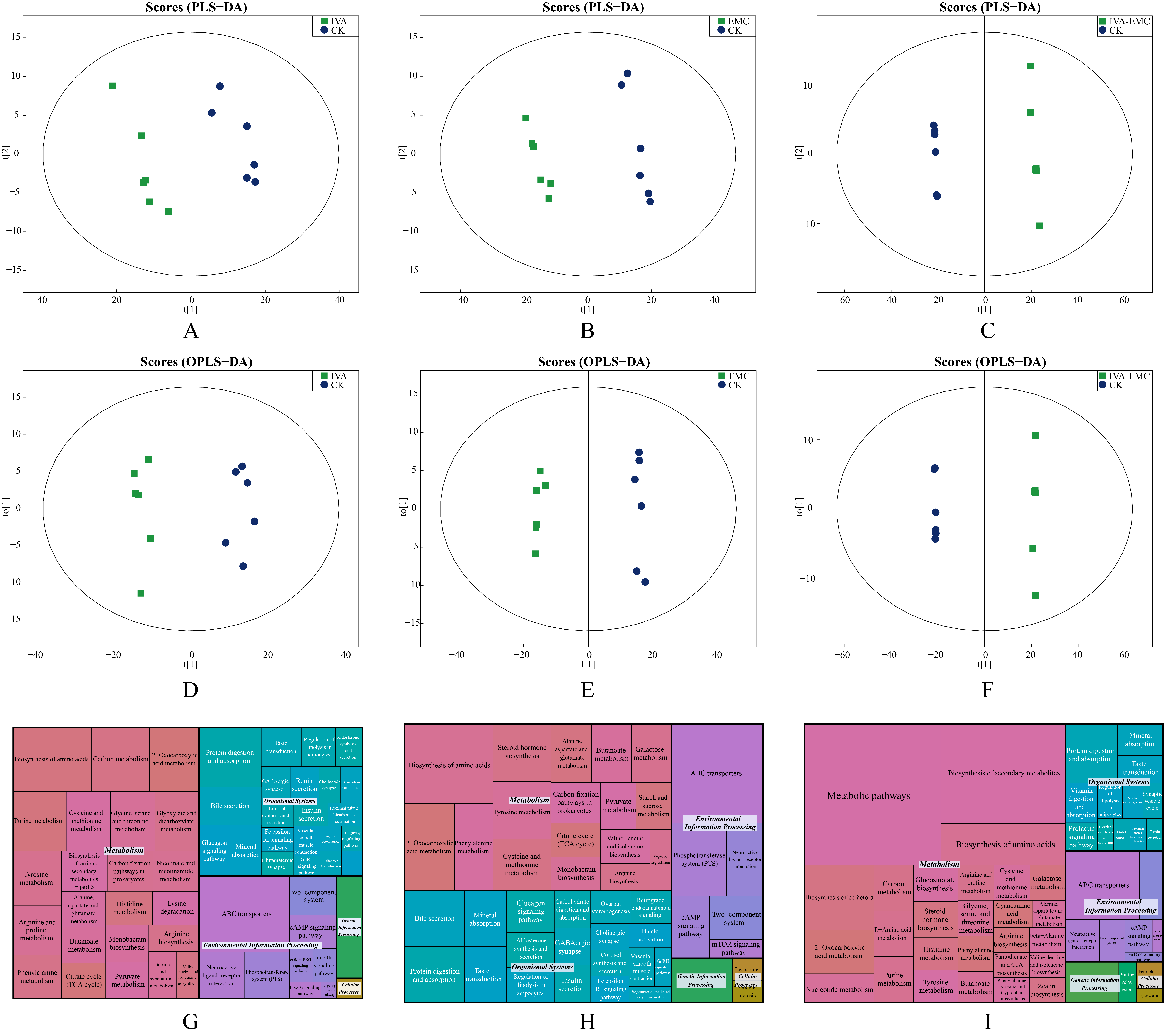
**

**Fig. S3.** Multivariate analysis and enrichment analysis of *P. destructans* mycelial metabolome after VOCs treatment. (A-C) PLS-DA score plots for the IVA, EMC and IVA-EMC groups, respectively. (D-F) OPLS-DA score plots for the IVA, EMC and IVA-EMC groups, respectively. (G-I) KEGG-enriched significant pathways in the IVA, EMC and IVA-EMC groups, respectively.

**

**

**Fig. S4.** Integrative transcriptomic and metabolomic analysis**.** (A-C) Correlation analysis of the top 20 DEGs and DEMs in the IVA, EMC, and IVA-EMC groups, respectively. (D-F) KEGG pathway annotation of DEGs and DEMs in the IVA, EMC, and IVA-EMC groups, respectively.

**Table S1.** Primers used for RT-qPCR.

| **Gene** | **Primers (5′-3′)** | **MW** | **GC Content (%)** | **TM (°C)** | **Transcript ID** |
| --- | --- | --- | --- | --- | --- |
| *CHS1*  *(VC83_06323)* | F: TAATCGTTTAGGTCCGCAAGG  R: GTCGTGGTGATAGCCTTGGTT | 6452  6500 | 47.6  52.4 | 58  60 | XM_024469925.1 |
| *SP2*  *(VC83_06062)* | F: GGCTTCAAGGGCTACCAGATC  R: GAGCATTAGCTTGGACCTTTCC | 6432  6702 | 57.1  50 | 61.9  60.1 | XM_024469668.1 |
| *ZWF1*  *(VC83_05048)* | F: CCAGGACGAAGATGGACAATG  R: CGACTCGTCCTCATCATATTGC | 6514  6622 | 52.4  50 | 60.1  60 | XM_024468673.1 |
| *SDH1*  *(VC83_04314)* | F: CAATCGCAAGACTTCGGAAAG  R: GATCAGGTCAAGGGCGAAGTA | 6449  6545 | 47.6  52.4 | 58  60 | XM_024467949.1 |
| *ATP5*  *(VC83_03795)* | F: GTCTATGTCAAGGACCCAAAGCT  R: AACTTGGTGCAGACACCTTCG | 7034  6407 | 47.8  52.4 | 60.2  60 | XM_024467438.1 |
| *PDE2*  *(VC83_00282)* | F: GAGGACGACGACGAGAATGAG  R: ATCACAGCAACTGGAAGCACC | 6579  6394 | 57.1  52.4 | 61.9  60 | XM_024463977.1 |
| *ADE13*  *(VC83_00579)* | F: GGAGGCCCTAGAGCAGATGAG  R: GCAGCTTGTTGCTCCGTAGTG | 6546  6445 | 61.9  57.1 | 63.9  61.9 | XM_024466669.1 |
| *MCM6*  *(VC83_05572)* | F: CTCCAGCAATCAGGCTTCTTC  R: TCGATAGAAGCTGGCCGATAT | 6318  6471 | 52.4  47.6 | 60  58 | XM_024471677.1 |
| *PKA1*  *(VC83_05871)* | F: AACAGTATGCGCCTTCACCTC  R: CAAGCTGTACTTGCCCTTGGT | 6327  6389 | 52.4  52.4 | 60  60 | XM_024469052.1 |
| *EFG1*  *(VC83_05289)* | F: TACCCGCTGAACGAACGATAC  R: CCGTCTTTGGCTTCTCAACAT | 6385  6324 | 52.4  47.6 | 60  58 | XM_024468911.1 |
| *CRZ1*  *(VC83_08501)* | F: TGTCCGGTCTAAACCAGCACC  R: TTCGCCGGTATGCAGTTTAGAG | 6352  6782 | 57.1  50 | 61.9  60.1 | XM_024472050.1 |
| *HSP98*  *(VC83_08137)* | F: AAAGCAAAGACCAGCAGCAAT  R: GCCGACCTAAGAATCTTGCTG | 6451  6407 | 42.9  52.4 | 56.1  60 | XM_024471698.1 |
| *HSP90*  *(VC83_08187)* | F: AGCAGACCGACTACCTCAACG  R: TGGCTTCTTGTCATCTCCCTC | 6370  6291 | 57.1  52.4 | 61.9  60 | XM_024464290.1 |

F, forward primer; R, reverse primer. *EFG1*, reference gene

**Table S2.** Fractional inhibitory concentration index values for combinations of IVA and EMC against *P. destructans*.

| **EMC\IVA** | **0.04** | **0.08** | **0.16** | **0.32** | **0.64** | **1.28** |
| --- | --- | --- | --- | --- | --- | --- |
| **0.13** | *0.125* | *0.188* | *0.313* | 0.563 | 1.063 | 2.063 |
| **0.26** | *0.188* | *0.250* | 0.375 | 0.625 | 1.125 | 2.125 |
| **0.52** | *0.313* | *0.375* | 0.500 | 0.750 | 1.250 | 2.250 |
| **1.04** | 0.563 | 0.625 | 0.750 | 1.000 | 1.500 | 2.500 |
| **2.08** | 1.063 | 1.125 | 1.250 | 1.500 | 2.000 | 3.000 |
| **4.16** | 2.063 | 2.125 | 2.250 | 2.500 | 3.000 | 4.000 |

Combinations that do not reach the MIC are indicated in italics.

**Table S3.** Overview of RNA-seq read quality.

| **Samples** | **Raw Reads** | **Clean Reads** | **N Ratio(%)** | **Q30 Ratio(%)** | **GC Ratio(%)** | **Total Map** |
| --- | --- | --- | --- | --- | --- | --- |
| CK-1 | 51075724 | 46720646 | 0.01% | 95.50% | 54.95% | 95.22% |
| CK-2 | 54838126 | 50806536 | 0.01% | 95.97% | 54.94% | 94.88% |
| CK-3 | 52237464 | 44257558 | 0.01% | 95.95% | 54.49% | 95.31% |
| IVA-1 | 45421426 | 39599834 | 0.01% | 95.69% | 54.70% | 95.53% |
| IVA-2 | 50879104 | 44510193 | 0.01% | 95.31% | 54.88% | 95.74% |
| IVA-3 | 60719320 | 53930382 | 0.01% | 95.81% | 55.24% | 95.91% |
| EMC-1 | 56069702 | 49629247 | 0.01% | 95.76% | 54.97% | 95.62% |
| EMC-2 | 60077770 | 53650880 | 0.01% | 95.87% | 55.05% | 95.97% |
| EMC-3 | 53590298 | 46721638 | 0.01% | 95.58% | 54.81% | 95.61% |
| IVA-EMC-1 | 40193736 | 37948293 | 0.26% | 95.95% | 56.29% | 96.24% |
| IVA-EMC-2 | 49286906 | 46482891 | 0.26% | 95.58% | 56.12% | 96.14% |
| IVA-EMC-3 | 51157508 | 48160381 | 0.26% | 95.52% | 56.11% | 95.93% |

**Table S4.** Annotation of 15 key genes using blastx in the SwissProt database (Lowest E-value considered).

| **Gene ID** | **K-value** | **Annotation** | **Gene name** | **Classification** |
| --- | --- | --- | --- | --- |
| VC83_07417 | 140 | Spore development regulator RYP2 | RYP2 | Transcription factor |
| VC83_02229 | 137 | NADH-cytochrome b5 reductase 2, | MCR1 | Structural gene |
| VC83_07050 | 137 | - | - | Uncharacterized protein |
| VC83_07536 | 136 | - | - | Uncharacterized protein |
| VC83_08500 | 133 | - | - | Uncharacterized protein |
| VC83_08501 | 133 | Transcriptional regulator CRZ1 | CRZ1 | Transcription factor |
| VC83_05679 | 133 | Ankyrin-3 | Ank3 | Uncharacterized protein |
| VC83_00940 | 133 | - | - | Uncharacterized protein |
| VC83_00665 | 130 | Putative RNA polymerase II subunit B1 CTD phosphatase rpap-2 | rpap-2 | Structural gene |
| VC83_03386 | 128 | E3 ubiquitin-protein ligase RSL1 | RSL1 | Structural gene |
| VC83_03691 | 128 | - | - | Uncharacterized protein |
| VC83_09495 | 126 | RNA polymerase-associated protein RTF1 | RTF1 | Transcription factor |
| VC83_07519 | 125 | GTPase-binding protein rid1 | rid1 | Structural gene |
| VC83_01468 | 124 | Glucose/galactose transporter | gluP | Structural gene |
| VC83_09042 | 122 | - | - | Uncharacterized protein |

**Table S5.** Expression of putative virulence genes in *P. destructans* following exposure to VOCs. Blastx used the lowest E-value in the SwissProt database, considering only E-values < 1E-4. Genes were considered significantly different if log_2_|fold change| >1 and adjusted *P*-value (Benjamini-Hochberg adjustment) < 0.05 on DESeq2 analyses.

|  |  |  | **Differentially expressed in *P. destructans* after exposure to:** | | |
| --- | --- | --- | --- | --- | --- |
| **Gene ID** | **Full Name** | **Blastx** | **IVA** | **EMC** | **IVA-EMC** |
| **Secreted Proteases** | | | | | |
| VC83_03247 | Probable extracellular serine carboxypeptidase | A1345_ARTBC | Down | Down | Down |
| VC83_06062 | Subtilisin-like protease 2 | - | Down | Down |  |
| VC83_06060 | Probable aspartic-type endopeptidase OPSB | OPSB_TRIVH |  | Up | Up |
| VC83_06748 | Putative aspergillopepsin A-like aspartic endopeptidase AFUA_2G15950 | Y5950_ASPFU |  | Down | Down |
| VC83_00241 | Aspergillopepsin-2 | PRTA_ASPNG |  | Up | Up |
| VC83_01226 | Secreted lipase ARB07186/07185 | LIP1_ARTBC |  | Down | Down |
| VC83_00947 | Secreted mono- and diacylglycerol lipase 1 | FGL1_GIBZE |  | Down | Down |
| VC83_04892 | Subtilisin-like protease 1 | - |  | Down |  |
| VC83_02385 | Zinc metalloprotease ZmpB | ZMPB_STRPN |  | Down |  |
| VC83_03800 | Disintegrin and metalloproteinase domain-containing protein B | ADMB_ASPFU |  | Down |  |
| VC83_05359 | Cysteine protease | - |  | Down |  |
| VC83_08633 | Threonine aspartase 1 | TASP1_HUMAN |  |  | Down |
| VC83_01361 | Major allergen Aspf2 | ALL2_ASPFU |  |  | Up |
| VC83_07090 | Serine protease | - |  |  | Up |
| VC83_01523 | Aspergillopepsin-1 | PEPA_ASPFN |  |  | Down |
| **Cell Wall Remodeling** | | | | | |
| VC83_06323 | Chitin synthase 1 | - | Down | Down |  |
| VC83_05759 | Chitin synthase 7 | - | Down | Down |  |
| VC83_07145 | Hydrolase 76 protein | - | Down | Down |  |
| VC83_03500 | Spherulin-1A | - | Down | Down |  |
| VC83_01854 | GPI inositol deacylase | - | Down | Down |  |
| VC83_08448 | Protein SUR7 | SUR7_CANAL | Down | Down |  |
| VC83_00788 | Chitinase 1 | CHI1_APHAL |  | Down | Up |
| VC83_07327 | Probable glucan endo-1,3-b-glucosidase eglC | EGLC_NEOFI |  | Down | Down |
| VC83_05475 | Cell wall alpha-1,3-glucan synthase ags1 | - | Down |  |  |
| VC83_09076 | Glucan 1,3-b-glucosidase | EXG1_COCCA |  | Down |  |
| VC83_00261 | Mannan endo-1,6-a-mannosidase DFG5 | DFG5_CANAL |  | Up |  |
| VC83_00837 | Secreted chitinase LysM12 | LYS12_PENEN |  | Down |  |
| VC83_02183 | Chitin synthase 2 | - |  |  | Up |
| VC83_02280 | Chitin biosynthesis protein chs5 | - |  |  | Up |
| VC83_06199 | Chitin synthase 2 | - |  |  | Up |
| VC83_07867 | Uncharacterized protein AFUA_6G02800 | YA280_ASPFU |  |  | Down |
| VC83_05292 | Cell wall mannoprotein CIS3 | CIS3_YEAS7 |  |  | Up |
| VC83_01650 | Hydrolase 76 protein | - |  |  | Down |
| VC83_00126 | Endochitinase A | CHIA1_EMENI |  |  | Down |
| VC83_04729 | Endochitinase 1 | CHI1_COCIM |  |  | Down |
| VC83_06494 | Endochitinase B1 | CHIB1_ASPFU |  |  | Down |
| VC83_08777 | Glycosylphosphatidylinositol (GPI) anchor assembly protein | - |  |  | Up |
| VC83_01831 | Glycosyl phosphatidyl inositol anchor synthesis | - |  |  | Down |
| VC83_03641 | GPI transamidase component | - |  |  | Up |
| VC83_00414 | Alpha 1,2 mannosyltransferase | - |  |  | Up |
| **Ion Homeostasis** | | | | | |
| VC83_01014 | Calcium-transporting ATPase 2 | ATC2_SCHPO | Down | Down | Down |
| VC83_01092 | Iron transport multicopper oxidase fetC | FETC_EPIFE | Up |  | Down |
| VC83_00191 | High-affinity copper transporter ctrA2 | CTRA2_ASPFU | Down |  | Down |
| VC83_01360 | Zinc-regulated transporter 1 | ZRT1_CANAL |  | Down | Down |
| VC83_01963 | Laccase-1 | LAC1_BOTFU |  | Down | Down |
| VC83_07026 | Na^+^ ATPase | - |  | Down |  |
| VC83_02763 | Na^+^ ATPase | - |  |  | Up |
| VC83_07127 | Na^+^ ATPase |  |  |  | Up |
| VC83_02764 | Na^+^ ATPase |  |  |  | Up |
| VC83_02762 | Sodium/potassium exporting P-type ATPase 2 | ATN2_YEAST |  |  | Up |
| VC83_06862 | Calcium-transporting ATPase 3 | ATC3_SCHPO |  | Down |  |
| VC83_00242 | Laccase-2 | LAC2_BOTFU |  |  | Down |
| VC83_07150 | Iron transport multicopper oxidase FET3 | FET3_GIBZE |  |  | Down |
| **Heat Shock Response** | | | | | |
| VC83_09034 | Unchar. J domain-containing protein C63.13 | YCJD_SCHPO | Down | Down | Down |
| VC83_01046 | Hsp70 chaperone | - |  | Down | Down |
| VC83_02466 | Uncharacterized protein C1711.08 | YNY8_SCHPO |  | Down | Down |
| VC83_06414 | Heat shock protein ssb1 | - |  | Up | Up |
| VC83_02553 | 30 kDa heat shock protein | HSP30_NEUCR |  |  | Down |
| VC83_07843 | hsp70 nucleotide exchange factor fes1 | - |  |  | Down |
| VC83_00970 | chaperone ATPase hsp78 | - |  |  | Down |
| VC83_00522 | Protein psi1 | PSI1_SCHPO |  |  | Down |
| VC83_01964 | adenyl-nucleotide exchange factor sse1 | - |  |  | Down |
| VC83_08137 | Heat shock protein hsp98 | - |  |  | Down |
| VC83_08187 | Heat shock protein 90 | - |  |  | Down |
| VC83_06435 | Hsp90 cochaperone | - |  |  | Down |
| VC83_05502 | 10 kDa heat shock protein | - |  |  | Down |
| **Other** | | | | | |
| VC83_01916 | Regulator of phospholipase D SRF1 | SRF1_YEAST | Down | Down | Up |
| VC83_00225 | Putative cryptochrome DASH, mitochondrial | CRYD_NEUCR |  | Down | Down |
| VC83_01624 | Leptomycin B resistance protein pmd1 | PMD1_SCHPO |  | Down | Down |
| VC83_03453 | Peptidase (Ustiloxin B biosynthesis protein) | USTQ_ASPFN |  | Down |  |
| VC83_06039 | Putative heme-binding peroxidase | CCPR2_ASPFU |  |  | Down |
| VC83_06307 | Squalene epoxidase | - |  |  | Down |
| VC83_03222 | GTP cyclohydrolase II | - |  |  | Down |
| VC83_06509 | Thioredoxin-disulfide reductase | - |  |  | Down |
| VC83_08771 | Probable transporter MCH5 | MCH5_YEAST |  |  | Down |
| VC83_04862 | DNA-dependent metalloprotease WSS1 homolog | WSS2_SCHPO |  |  | Down |
| VC83_02543 | Carboxypeptidase cpdS | PEPS_ASPPH |  |  | Up |
| VC83_05858 | Ubiquitin carboxyl-terminal hydrolase 34 | UBP34_HUMAN |  |  | Down |
| VC83_06276 | Tripeptidyl-peptidase SED4 | SED4_ASPFU |  |  | Down |
| VC83_01661 | Lysophosphatidylserine lipase ABHD12 | ABD12_XENTR |  |  | Down |

**Table S6.** Lists of significantly enriched pathways for DEGs in the IVA, EMC, and IVA-EMC Groups, respectively (*P* <0.05)

| **Pathway ID** | **KEGG-A-class** | **Pathway** | **Significant** | **Annotated** | ***P*-value** |
| --- | --- | --- | --- | --- | --- |
| ko05171 | Human Diseases | Coronavirus disease - COVID-19 | 31/163 | 83/1946 | 3.89E-14 |
| ko03010 | Genetic Information Processing | Ribosome | 31/163 | 108/1946 | 1.23E-10 |
| ko04016 | Environmental Information Processing | MAPK signaling pathway - plant | 4/163 | 6/1946 | 0.000623366 |
| ko00290 | Metabolism | Valine, leucine and isoleucine biosynthesis | 5/163 | 16/1946 | 0.00791244 |
| ko00630 | Metabolism | Glyoxylate and dicarboxylate metabolism | 6/163 | 26/1946 | 0.017648203 |
| ko00230 | Metabolism | Purine metabolism | 9/163 | 49/1946 | 0.017904145 |
| ko02025 | Cellular Processes | Biofilm formation - Pseudomonas aeruginosa | 2/163 | 3/1946 | 0.019774045 |
| ko00521 | Metabolism | Streptomycin biosynthesis | 3/163 | 8/1946 | 0.023587736 |
| ko00260 | Metabolism | Glycine, serine and threonine metabolism | 7/163 | 36/1946 | 0.026538304 |
| ko04626 | Organismal Systems | Plant-pathogen interaction | 2/163 | 4/1946 | 0.037379669 |
| ko04213 | Organismal Systems | Longevity regulating pathway - multiple species | 6/163 | 31/1946 | 0.039850113 |

*Significantly enriched pathways among DEGs in the IVA treatment group.*

| **Pathway ID** | **KEGG-A-class** | **Pathway** | **Significant** | **Annotated** | ***P*-value** |
| --- | --- | --- | --- | --- | --- |
| ko05171 | Human Diseases | Coronavirus disease - COVID-19 | 75/451 | 83/1946 | 9.97E-41 |
| ko03010 | Genetic Information Processing | Ribosome | 78/451 | 108/1946 | 6.91E-29 |
| ko05415 | Human Diseases | Diabetic cardiomyopathy | 34/451 | 64/1946 | 1.20E-07 |
| ko05208 | Human Diseases | Chemical carcinogenesis - reactive oxygen species | 36/451 | 74/1946 | 8.88E-07 |
| ko04714 | Organismal Systems | Thermogenesis | 34/451 | 69/1946 | 1.25E-06 |
| ko00190 | Metabolism | Oxidative phosphorylation | 36/451 | 76/1946 | 2.01E-06 |
| ko04932 | Human Diseases | Non-alcoholic fatty liver disease | 26/451 | 49/1946 | 4.11E-06 |
| ko05020 | Human Diseases | Prion disease | 44/451 | 105/1946 | 8.82E-06 |
| ko05010 | Human Diseases | Alzheimer disease | 43/451 | 110/1946 | 8.61E-05 |
| ko05012 | Human Diseases | Parkinson disease | 41/451 | 105/1946 | 0.000130729 |
| ko04260 | Organismal Systems | Cardiac muscle contraction | 11/451 | 17/1946 | 0.000288591 |
| ko05022 | Human Diseases | Pathways of neurodegeneration - multiple diseases | 46/451 | 130/1946 | 0.000743251 |
| ko05014 | Human Diseases | Amyotrophic lateral sclerosis | 44/451 | 132/1946 | 0.00380334 |
| ko00680 | Metabolism | Methane metabolism | 10/451 | 19/1946 | 0.004821812 |
| ko05016 | Human Diseases | Huntington disease | 43/451 | 130/1946 | 0.004953507 |
| ko05145 | Human Diseases | Toxoplasmosis | 6/451 | 9/1946 | 0.006601074 |
| ko05417 | Human Diseases | Lipid and atherosclerosis | 12/451 | 27/1946 | 0.011378073 |
| ko04392 | Environmental Information Processing | Hippo signaling pathway - multiple species | 4/451 | 5/1946 | 0.011648433 |
| ko00020 | Metabolism | Citrate cycle (TCA cycle) | 11/451 | 24/1946 | 0.011654574 |
| ko05163 | Human Diseases | Human cytomegalovirus infection | 10/451 | 22/1946 | 0.017109392 |
| ko05167 | Human Diseases | Kaposi sarcoma-associated herpesvirus infection | 8/451 | 17/1946 | 0.025680633 |
| ko00010 | Metabolism | Glycolysis / Gluconeogenesis | 13/451 | 33/1946 | 0.026306014 |
| ko00071 | Metabolism | Fatty acid degradation | 9/451 | 21/1946 | 0.035271286 |
| ko04360 | Organismal Systems | Axon guidance | 7/451 | 15/1946 | 0.038296743 |

*Significantly enriched pathways among DEGs in the EMC treatment group.*

| **Pathway ID** | **KEGG-A-class** | **Pathway** | **Significant** | **Annotated** | ***P*-value** |
| --- | --- | --- | --- | --- | --- |
| ko05171 | Human Diseases | Coronavirus disease - COVID-19 | 76/622 | 83/1946 | 3.20E-31 |
| ko03010 | Genetic Information Processing | Ribosome | 80/622 | 108/1946 | 2.64E-20 |
| ko00630 | Metabolism | Glyoxylate and dicarboxylate metabolism | 18/622 | 26/1946 | 9.65E-05 |
| ko00020 | Metabolism | Citrate cycle (TCA cycle) | 17/622 | 24/1946 | 9.74E-05 |
| ko00500 | Metabolism | Starch and sucrose metabolism | 22/622 | 38/1946 | 0.000770605 |
| ko00680 | Metabolism | Methane metabolism | 13/622 | 19/1946 | 0.001146898 |
| ko00620 | Metabolism | Pyruvate metabolism | 21/622 | 37/1946 | 0.001444957 |
| ko00010 | Metabolism | Glycolysis / Gluconeogenesis | 19/622 | 33/1946 | 0.001936161 |
| ko00720 | Metabolism | Carbon fixation pathways in prokaryotes | 8/622 | 11/1946 | 0.006496526 |
| ko04016 | Environmental Information Processing | MAPK signaling pathway - plant | 5/622 | 6/1946 | 0.01455362 |
| ko00260 | Metabolism | Glycine, serine and threonine metabolism | 18/622 | 36/1946 | 0.017512198 |
| ko00920 | Metabolism | Sulfur metabolism | 8/622 | 13/1946 | 0.026500593 |
| ko00440 | Metabolism | Phosphonate and phosphinate metabolism | 3/622 | 3/1946 | 0.0325473 |
| ko04940 | Human Diseases | Type I diabetes mellitus | 3/622 | 3/1946 | 0.0325473 |
| ko04612 | Organismal Systems | Antigen processing and presentation | 5/622 | 7/1946 | 0.037577785 |
| ko00710 | Metabolism | Carbon fixation in photosynthetic organisms | 10/622 | 19/1946 | 0.048700285 |

*Significantly enriched pathways among DEGs in the IVA-EMC treatment group.*

**Table S7.** List of metabolic pathways matching with shared DEGs identified across all treatment groups (*P* <0.05).

| **Pathway ID** | **KEGG_A_class** | **KEGG_B_class** | **Pathway** | **Out (83)** | **All (1939)** | ***P*-value** |
| --- | --- | --- | --- | --- | --- | --- |
| ko05171 | Human Diseases | Infectious disease: viral | Coronavirus disease - COVID-19 | 31 | 83 | 1.10E-23 |
| ko03010 | Genetic Information Processing | Translation | Ribosome | 31 | 108 | 1.03E-19 |
| ko00260 | Metabolism | Amino acid metabolism | Glycine, serine and threonine metabolism | 6 | 36 | 0.00355895 |
| ko00920 | Metabolism | Energy metabolism | Sulfur metabolism | 3 | 13 | 0.01585131 |
| ko01200 | Metabolism | Global and overview maps | Carbon metabolism | 9 | 95 | 0.01792281 |
| ko00630 | Metabolism | Carbohydrate metabolism | Glyoxylate and dicarboxylate metabolism | 4 | 26 | 0.02272397 |
| ko04016 | Environmental Information Processing | Signal transduction | MAPK signaling pathway - plant | 2 | 6 | 0.02427588 |
| ko04622 | Organismal Systems | Immune system | RIG-I-like receptor signaling pathway | 2 | 7 | 0.03305105 |
| ko00930 | Metabolism | Xenobiotics biodegradation and metabolism | Caprolactam degradation | 1 | 1 | 0.04280557 |
| ko01210 | Metabolism | Global and overview maps | 2-Oxocarboxylic acid metabolism | 4 | 33 | 0.04963159 |

**Table S8.** Common KEGG enrichment pathways information for all treatment groups. (*P* <0.05)

| **Pathway** | **KEGG_A_class** | **KEGG_B_class** | **Pathway** | ***P-*(IVA)** | ***P*-(EMC)** | ***P*-(IVA-EMC)** |
| --- | --- | --- | --- | --- | --- | --- |
| ko02010 | Environmental Information Processing | Membrane transport | ABC transporters | 2.21872E-10 | 1.05783E-09 | 4.75569E-11 |
| ko04974 | Organismal Systems | Digestive system | Protein digestion and absorption | 8.71628E-09 | 0.000113267 | 1.97657E-11 |
| ko01230 | Metabolism | Global and overview maps | Biosynthesis of amino acids | 3.45802E-06 | 2.01846E-06 | 1.11763E-12 |
| ko00970 | Genetic Information Processing | Translation | Aminoacyl-tRNA biosynthesis | 8.37022E-05 | 0.004879249 | 4.6616E-06 |
| ko04923 | Organismal Systems | Endocrine system | Regulation of lipolysis in adipocytes | 0.000252289 | 0.000493322 | 0.000354163 |
| ko04150 | Environmental Information Processing | Signal transduction | mTOR signaling pathway | 0.000346587 | 0.00053156 | 0.013065654 |
| ko00270 | Metabolism | Amino acid metabolism | Cysteine and methionine metabolism | 0.000437011 | 0.004806533 | 0.041614015 |
| ko04024 | Environmental Information Processing | Signal transduction | cAMP signaling pathway | 0.000667081 | 0.000201565 | 0.006069596 |
| ko00250 | Metabolism | Amino acid metabolism | Alanine, aspartate and glutamate metabolism | 0.001266854 | 0.000433997 | 0.009975798 |
| ko04978 | Organismal Systems | Digestive system | Mineral absorption | 0.001538152 | 0.000547038 | 4.74093E-05 |
| Ko04080 | Environmental Information Processing | Signaling molecules and interaction | Neuroactive ligand-receptor interaction | 0.002006861 | 0.004879249 | 0.003568887 |
| Ko00350 | Metabolism | Amino acid metabolism | Tyrosine metabolism | 0.002378724 | 0.00662752 | 0.033554677 |
| Ko04742 | Organismal Systems | Sensory system | Taste transduction | 0.002620591 | 0.001031638 | 0.000675388 |
| Ko00220 | Metabolism | Amino acid metabolism | Arginine biosynthesis | 0.003040787 | 0.028762912 | 0.004160346 |
| Ko02060 | Environmental Information Processing | Membrane transport | Phosphotransferase system (PTS) | 0.003630979 | 2.62519E-05 | 0.00146697 |
| Ko00360 | Metabolism | Amino acid metabolism | Phenylalanine metabolism | 0.00500701 | 0.000905546 | 0.008545722 |
| Ko02020 | Environmental Information Processing | Signal transduction | Two-component system | 0.008936611 | 0.018652827 | 0.041112106 |
| Ko00650 | Metabolism | Carbohydrate metabolism | Butanoate metabolism | 0.010429133 | 0.00530051 | 0.024407947 |
| Ko04927 | Organismal Systems | Endocrine system | Cortisol synthesis and secretion | 0.014575352 | 0.02142911 | 0.017709826 |
| Ko01210 | Metabolism | Global and overview maps | 2-Oxocarboxylic acid metabolism | 0.016795013 | 0.021051835 | 0.000523189 |
| Ko00290 | Metabolism | Amino acid metabolism | Valine, leucine and isoleucine biosynthesis | 0.017939094 | 0.005641571 | 0.004160346 |

**Table S9.** List of top 15 metabolic pathways matching with shared DEMs identified across all treatment groups.

| **Pathway ID** | **KEGG_A_class** | **KEGG_B_class** | **Pathway** | **Out (83)** | **All (1939)** | ***P*-value** |
| --- | --- | --- | --- | --- | --- | --- |
| ko01210 | Metabolism | Global and overview maps | 2-Oxocarboxylic acid metabolism | 5 | 29 | 0.02429148 |
| ko02010 | Environmental Information Processing | Membrane transport | ABC transporters | 8 | 66 | 0.03398528 |
| ko00780 | Metabolism | Metabolism of cofactors and vitamins | Biotin metabolism | 2 | 6 | 0.04529255 |
| ko00350 | Metabolism | Amino acid metabolism | Tyrosine metabolism | 3 | 15 | 0.05509774 |
| ko01053 | Metabolism | Metabolism of terpenoids and polyketides | Biosynthesis of siderophore group nonribosomal peptides | 1 | 1 | 0.06017699 |
| ko04625 | Organismal Systems | Immune system | C-type lectin receptor signaling pathway | 1 | 1 | 0.06017699 |
| ko00500 | Metabolism | Carbohydrate metabolism | Starch and sucrose metabolism | 2 | 7 | 0.06104252 |
| ko05207 | Human Diseases | Cancer: overview | Chemical carcinogenesis - receptor activation | 2 | 7 | 0.06104252 |
| ko00996 | Metabolism | Biosynthesis of other secondary metabolites | Biosynthesis of various alkaloids | 2 | 10 | 0.1168047 |
| ko00401 | Metabolism | Biosynthesis of other secondary metabolites | Novobiocin biosynthesis | 1 | 2 | 0.116833 |
| ko01055 | Metabolism | Metabolism of terpenoids and polyketides | Biosynthesis of vancomycin group antibiotics | 1 | 2 | 0.116833 |
| ko04114 | Cellular Processes | Cell growth and death | Oocyte meiosis | 1 | 2 | 0.116833 |
| ko04914 | Organismal Systems | Endocrine system | Progesterone-mediated oocyte maturation | 1 | 2 | 0.116833 |
| ko05224 | Human Diseases | Cancer: specific types | Breast cancer | 1 | 2 | 0.116833 |
| ko00521 | Metabolism | Biosynthesis of other secondary metabolites | Streptomycin biosynthesis | 1 | 3 | 0.1701681 |

**Table S10.** Abbreviations of **Fig. 5** and **Fig. 6**.

| Abbreviations | Full name of abbreviations |
| --- | --- |
| DEGs | Differentially expressed genes |
| DEMs | Differentially expressed metabolites |
| PRPP | 5-Phosphoribosyl 1-pyrophosphate |
| SAICAR | 1-(5'-Phosphoribosyl)-5-amino-4-(N-succinocarboxamide)-imidazole |
| CAIR | 5'-Phosphoribosyl-5-aminoimidazole carboxylate |
| FAICAR | 5'-Phosphoribosyl-5-formamido-4-imidazolecarboxamide |
| IMP | Inosine monophosphate |
| AMP | Adenosine 5'-monophosphate |
| dAMP | Deoxyadenosine monophosphate |
| ADP | Adenosine 5'-diphosphate |
| dADP | 2'-Deoxyadenosine 5'-diphosphate |
| ATP | Adenosine 5'-triphosphate |
| dATP, | Deoxyadenosine triphosphate |
| 3',5'-Cyclic AMP | Adenosine 3',5'-phosphate |
| XMP | Xanthosine 5'-phosphate |
| 3',5'-Cyclic GMP | Guanosine 3',5'-cyclic phosphate |
| GMP | guanosine monophosphate |
| dGMP | Deoxyguanosine monophosphate |
| GDP | Guanosine diphosphate |
| dGDP | 2'-Deoxyguanosine 5'-diphosphate |
| GTP | Guanosine 5'-triphosphate |
| dGTP | Deoxyguanosine triphosphate |
| DNA | Deoxyribonucleic acid |
| RNA | Ribonucleic acid |
| ADE13 | adenylosuccinase ade13 |
| ADE1 | Bifunctional purine biosynthetic protein ade1 |
| ADE17 | Bifunctional phosphoribosylaminoimidazolecarboxamide formyltransferase/IMP cyclohydrolase |
| PDE2 | 3',5'-cyclic-nucleotide phosphodiesterase |
| MCM6 | DNA replication licensing factor MCM2 |
| MCM2 | DNA replication licensing factor MCM2 |
| MCM4 | DNA replication licensing factor MCM4 |
| MCM7 | DNA replication licensing factor MCM7 |
| POL30 | proliferating cell nuclear antigen |
| FEN1 | Elongation of fatty acids protein 2 |
| POL1 | DNA-directed DNA polymerase alpha catalytic subunit pol1 |
| POL12 | DNA-directed DNA polymerase alpha subunit pol12 |
| RFA2 | replication factor A2 |
| DNA2 | DNA replication ATP-dependent helicase/nuclease Dna2 |
| PRI1 | DNA primase small subunit |
| RPA3 | replication factor A3 |
| CYR1_2 | cysteinyl-tRNA synthetase |
| CYR1_1 | cysteinyl-tRNA synthetase |
| NDK1 | nucleoside diphosphate kinase |
| ADK1 | Adenylate kinase |
| GUA1 | GMP synthase (glutamine-hydrolyzing) |
| RNASEH2A | ribonuclease H2 subunit A |
| FAA4 | long-chain fatty acid-CoA ligase |
| POX1 | fatty-acyl coenzyme A oxidase |
| echA | enoyl-CoA hydratase |
| ERG10 | erg10, acetyl-CoA C-acetyltransferase |
| GUT1 | Glycerol kinase |
| galM | aldose 1-epimerase |
| GLK1_1 | glucokinase |
| HXK1 | hexokinase A |
| PGI1 | glucose-6-phosphate isomerase |
| PFK1 | 6-phosphofructokinase, alpha subunit |
| TPI1 | triosephosphate isomerase |
| FBA1 | Fructose-bisphosphate aldolase 1 |
| PGK1 | phosphoglycerate kinase |
| ENO1 | phosphopyruvate hydratase |
| gplm | 2,3-bisphosphoglycerate-independent phosphoglycerate mutase |
| PYK1 | Pyruvate kinase |
| PDB1 | pyruvate dehydrogenase E1, beta subunit |
| ZWF1 | Glucose-6-phosphate 1-dehydrogenase |
| GND1 | phosphogluconate dehydrogenase (decarboxylating) gnd1 |
| TKL1_2 | Transketolase |
| TKL1_1 | Transketolase |
| xfp | xylulose-5-phosphate/fructose-6-phosphate phosphoketolase |
| ARO8_2 | Aromatic/aminoadipate aminotransferase 1 |
| HPD | 4-hydroxyphenylpyruvate dioxygenase |
| maiA | maleylacetoacetate isomerase |
| FAH | fumarylacetoacetase |
| HGD | homogentisate 1,2-dioxygenase |
| ASS1 | Adenylosuccinate synthetase |
| CAR1 | arginase |
| ARG4 | argininosuccinate lyase |
| ACO1 | Aconitate hydratase mitochondrial |
| MDH1 | Malate dehydrogenase, cytoplasmic |
| FUM1 | fumarase fum1 |
| TCA8,9 | Succinate--CoA ligase [ADP-forming] subunit alpha, beta, mitochondrial |
| KGD1,2 | 2-oxoglutarate dehydrogenase complex E1,2 component |
| ACL2 | glutathione synthetase ATP-binding domain-like protein |
| ACL1 | citrate synthase |
| CIT1 | citrate (Si)-synthase |
| CS2 | citrate synthase |
| CoQ | Coenzyme Q |
| NADH | Nicotinamide adenine dinucleotide (reduced form) |
| NAD⁺ | Nicotinamide adenine dinucleotide (oxidized form) |
| FAD / FADH_2_ | Flavin adenine dinucleotide (oxidized/reduced form) |
| Cyt c | Cytochrome c |
| SDH2 | succinate dehydrogenase complex, subunit B |
| SDH1 | succinate dehydrogenase flavoprotein subunit |
| SDH3 | succinate dehydrogenase (ubiquinone) cytochrome b560 subunit |
| ATP… | ATP synthase subunits |
| NAD5 | NADH-ubiquinone oxidoreductase chain 5 |
| COX… | Cytochrome c oxidase subunits |
| NDUF… | NADH dehydrogenase |
| NUO… | NADH-quinone oxidoreductase subunit |
| QCR… | ubiquinol-cytochrome c reductase core subunit |
| VMA1,7,5 | H(+)-transporting V1 sector ATPase subunit A,F,C |
| PMA1 | plasma membrane H+-ATPase |
| VMA7 | H(+)-transporting V1 sector ATPase subunit F |
| UQCRFS1 | ubiquinol-cytochrome c reductase iron-sulfur subunit |
| Pi | Inorganic Phosphate |
